# The Effects of Naringin on Antioxidant Function, Intestinal Barrier and Immune Response in Broilers Challenged with Lipopolysaccharide

**DOI:** 10.3390/ani15233367

**Published:** 2025-11-21

**Authors:** Ling Yang, Lianwei Tang, Shuangshuang Guo, Lei Wang, Yongqing Hou

**Affiliations:** 1 Engineering Research Center of Feed Protein Resources on Agricultural By-Products, Ministry of Education, Hubei Key Laboratory of Animal Nutrition and Feed Science, Wuhan Polytechnic University, Wuhan 430023, China; 2 Hubei Horwath Biotechnology Co., Ltd., Xianning 437000, China

**Keywords:** naringin, antioxidant function, intestinal barrier, immune response, broiler

## Abstract

This study aimed to investigate the effects of naringin (NG) on growth performance, antioxidant status, intestinal barrier function, and immune stress in broilers challenged with lipopolysaccharide (LPS). A total of 144 one-day-old Ross 308 broiler chicks were randomly allocated into 2 treatment groups, with six replicates per group and 12 birds per replicate. The groups consisted of a control group (fed a basal diet) and an NG group (fed the basal diet supplemented with 200 mg/kg NG). The trial lasted 21 days; to evaluate the impact of NG on the late-stage growth performance of broilers, the rearing trial continued until 35 days. Body weight was recorded on days 21 and 35 to evaluate growth performance. A 2 × 2 factorial design was implemented in which broilers were challenged with or without LPS, and their diets were supplemented with or without NG. On day 21, two birds per replicate were intraperitoneally injected with 0.5 mg/kg body weight (BW) LPS, while another two birds received an equal volume of saline, in each group of 12 birds. Birds were slaughtered 3 h post-injection for sample collection. Dietary NG supplementation had no significant effect on growth performance (*p* > 0.05). However, NG markedly improved plasma liver function markers following LPS challenge (*p* < 0.05). NG significantly increased hepatic glutathione peroxidase (GSH-Px) activity, duodenal and ileal catalase (CAT) activity, and ileal GSH-Px activity (*p* < 0.05) while reducing malondialdehyde (MDA) levels in the liver, duodenum, jejunum, and ileum, as well as hydrogen peroxide (H_2_O_2_) levels in the jejunum and ileum (*p* < 0.05). Dietary NG supplementation significantly upregulated the relative mRNA expression of *Toll-like receptor 4 (TLR4)* and *interferon-gamma (IFN-γ)* in the liver (*p* < 0.05) and attenuated the LPS-induced upregulation of *tumor necrosis factor-alpha (TNF-α)* mRNA expression in the jejunum (*p* < 0.05). NG reduced serum diamine oxidase (DAO) levels (*p* < 0.05), increased villus height in the duodenum and jejunum (*p* < 0.05), and increased the villus-height-to-crypt-depth (VH/CD) ratio in the jejunum (*p* < 0.05). Moreover, NG markedly upregulated the relative mRNA expression of *zonula occludens-1 (ZO-1)* and *occludin* in the duodenum while drastically downregulating the relative expression of *mucin-2* in the duodenum and ileum (*p* < 0.05). NG further reduced *mucin-2* expression in the jejunum and mitigated the LPS-induced downregulation of *ZO-1* in the ileum (*p* < 0.05). In addition, NG significantly upregulated the expression of *X-linked inhibitor of apoptosis protein (XIAP)* and *B-cell lymphoma 2 (Bcl-2)* and downregulated the expression of *matrix metalloproteinase-13 (MMP-13)* in the liver of LPS-challenged broilers (*p* < 0.05). Conclusions: Dietary NG supplementation alleviated LPS-induced intestinal and hepatic injury in broilers. NG attenuated the adverse effects of LPS challenge on intestinal barrier function and enhanced antioxidant capacity in broilers by modulating intestinal tight junction expression and antioxidant enzyme activity. NG may serve as a promising eco-friendly additive to enhance resilience against immune stress in broilers.

## Simple Summary

Immune stress is common in modern intensive, high-density poultry production systems and poses a major challenge to the healthy and sustainable development of the broiler industry. Its effects on intestinal function are particularly pronounced. Immune stress disrupts the integrity of the intestinal mucosal barrier, reduces digestive enzyme activity, impairs nutrient absorption, and leads to intestinal dysbiosis and systemic inflammation. Naringin (NG) is a natural flavonoid found in citrus peels such as pomelo, orange, and tangerine. It has been reported to possess antimicrobial, anti-inflammatory, antioxidant, hepatoprotective, and lipid metabolism-regulating properties. As a novel, eco-friendly feed additive, NG shows strong potential for application in animal production. This study investigated the effects of NG on antioxidant function, intestinal barrier and immune response in broilers challenged with lipopolysaccharide (LPS). The results demonstrated that dietary NG supplementation alleviated LPS-induced inflammation. By regulating tight junction expression and enhancing antioxidant enzyme activity, NG improved barrier integrity, immune function, and antioxidant capacity in LPS-challenged broilers. These findings provide a scientific basis for the potential use of NG in broiler production.

## 1. Introduction

Immune stress is common in modern intensive, high-density farming systems and hinders the healthy and sustainable development of the broiler industry, causing substantial economic losses annually. Immune stress refers to the physiological process whereby animals, in response to stimuli such as pathogen infection, vaccination, or environmental changes, activate their immune system and trigger non-specific physiological responses. These responses are often accompanied by cytokine release, metabolic disturbances, and reduced growth performance [1,2]. Among the consequences, the adverse impact on intestinal function is particularly critical. Studies have demonstrated that immune stress impairs the intestinal barrier integrity and significantly reduces digestive enzyme activity, leading to decreased nutrient absorption efficiency, while simultaneously inducing gut microbiota dysbiosis and systemic inflammatory responses [3,4]. Traditionally, antibiotics were added to the feed to address this issue. However, with increasing global concerns regarding food safety and quality, many countries have banned antibiotic use in animal feed. Consequently, bioactive plant-derived compounds, characterized by stable composition and high bioactivity, have emerged as a promising focus in the search for effective antibiotic alternatives.

Naringin (NG), a natural flavonoid and secondary metabolite predominantly found in the peels of citrus fruits such as pomelo, tangerine, and orange, has attracted considerable attention. NG exhibits multiple beneficial biological functions and possesses the advantages of non-toxicity, environmental friendliness, and sustainability, making it a promising candidate as a novel green feed additive in livestock production [5]. Liu et al. [6] showed that NG reduces pro-inflammatory cytokine release by inhibiting LPS-induced NF-κB and MAPK signaling pathways. Zhang et al. [7] reported that NG elevates NADPH and GSH levels, decreases peroxynitrite levels, inhibits the expression of Pin1 (a pro-oxidative protein), and alleviates oxidative stress. Furthermore, In an animal model of sepsis-induced intestinal injury, naringenin treatment significantly suppressed M1 macrophage polarization and promoted M2 macrophage polarization by inhibiting the STAT1 signaling pathway, thereby alleviating sepsis-induced intestinal injury [8].

Lipopolysaccharide (LPS), a major component of the outer membrane of Gram-negative bacteria (e.g., *Escherichia coli* and *Salmonella*), consists of lipid A (the toxic moiety), O-antigen, and core polysaccharides [9]. LPS binds to the CD14/TLR4/MD2 receptor complex on various cell types, triggering immunological stress, disrupting tight junctions in intestinal epithelial cells, compromising gut integrity, and inducing digestive disorders, growth retardation, and disease susceptibility, ultimately diminishing production efficiency [10,11]. The LPS-induced immune stress model exhibits high stability due to its well-defined pro-inflammatory mechanism, in which intracellular signaling cascades activate the key transcription factor NF-κB, which translocates to the nucleus, where it promotes the synthesis and release of inflammatory cytokines (e.g., TNF-α, IL-6, and IL-1β). These mediators infiltrate target tissues, causing lipid metabolism dysregulation and tissue damage [12,13]. Luo et al. [14] reported that NG alleviated LPS-induced intestinal barrier damage in mice by inhibiting inflammatory factors, improving antioxidant function, and preserving intestinal tight junction integrity.

Based on the findings above, this study aimed to investigate the effects of NG on the growth performance of broilers. By establishing an LPS-induced immune stress model, it explores the impact of naringin on antioxidant function, intestinal barrier, and immune response in broilers. These findings provide a scientific basis for the practical use of NG as a feed additive in poultry farming.

## 2. Materials and Methods

### 2.1. Animals and Experimental Design

All experimental procedures used were approved by the Institutional Animal Care and Use Committee of Wuhan Polytechnic University (protocol code: WPU202404003).

A total of 144 one-day-old Ross 308 broiler chicks with similar initial body weights were obtained from a commercial hatchery and randomly assigned to two groups. Each group consisted of six replicates with 12 birds per replicate (six males and six females, merged analysis of gender data). Birds in the control (CON) group were fed a corn–soybean meal basal diet, while birds in the NG group received the basal diet supplemented with 200 mg/kg NG. According to our previous study, the addition of 200 mg/kg NG in the basal diet could improve the growth performance, increase immunity, and antioxidant capacity of broilers. Therefore, the supplementation dose of NG in the diet was 200 mg/kg in the current experiment. The basal diet was formulated according to the NY/T33-2004 (Feeding standard of chicken, Beijing, China, 2004) recommendations for broilers, and its ingredient composition and calculated nutrient levels are presented in Table 1. NG (purity ≥ 95%, tested before the experiment) was provided by Hubei Horwath Biotechnology Co. (Xianning, China). The trial lasted 21 days, with slaughter sampling conducted at 21 days. To evaluate the impact of NG on the late-stage growth performance of broilers, the feeding trial lasted for 35 days. Broilers had ad libitum access to feed and water, and a 24 h light regime was implemented. The room temperature was maintained at 32~34 °C during the first week, then reduced by 3 °C per week until reaching 22 °C, which was maintained thereafter. Record the relative humidity daily using a hygrometer. Relative humidity was between 50~60% throughout the experiment.

### 2.2. Sample Collection

A 2 × 2 factorial design was implemented in which broilers were challenged with or without LPS, and their diets were supplemented with or without NG. At 21 days of age, after 8 h of withholding feed, four broilers were randomly selected from each replicate. Two birds (one male and one female) were intraperitoneally injected with LPS (0.5 mg/kg body weight), while the other two received an equal volume of sterile saline, in each group of 12 birds (six males and six females). Three hours after injection, 2 mL of blood was collected from the wing vein into heparinized vacuum tubes. Samples were centrifuged at 3000 r/min for 10 min at 4 °C, and plasma was separated, aliquoted into EP tubes, and stored at −80 °C for further analysis. Plasma biochemical parameters were analyzed on the day of blood collection, while plasma antioxidant parameters were analyzed within 2 weeks of storage. Birds were then euthanized by cervical dislocation. The thymus, spleen, bursa of Fabricius, and liver were excised and weighed. Liver samples were snap-frozen in liquid nitrogen and stored at −80 °C. Segments of the duodenum, jejunum, and ileum (≈1 cm each, mid-segment) were fixed in 4% paraformaldehyde. Intestinal mucosa scrapings were collected, frozen in liquid nitrogen, and stored at −80 °C for subsequent analysis.

### 2.3. Growth Performance

Broilers in each replicate were weighed at 21 and 35 days of age, all birds in each repetition were weighed together, and feed intake was recorded during days 1–21, 21–35, and 1–35. Average body weight (BW), average daily gain (ADG), average daily feed intake (ADFI), and feed conversion ratio (FCR) were calculated per replicate.

### 2.4. Plasma Biochemical Parameters

Plasma biochemical parameters were measured using a HITACHI Automatic Analyzer (Hitachi Ltd., Tokyo, Japan). The following indicators were determined: total protein (TP), albumin (ALB), globulin (GLB), total bilirubin (TB), aspartate aminotransferase (AST), alanine aminotransferase (ALT), alkaline phosphatase (ALP), γ-glutamyl transpeptidase (GGT), triglycerides (TG), total cholesterol (TC), high-density lipoprotein (HDL), low-density lipoprotein (LDL), and lactate dehydrogenase (LDH).

### 2.5. Organ Index

We weighed and recorded the liver, thymus, spleen, and bursa of Fabricius. We calculated the organ index using the following formula: Organ Index (g/kg) = Organ Weight/Live Weight.

### 2.6. Antioxidant Status

We took approximately 1 g of liver, duodenum, jejunum, and ileum tissue, removing any fat and connective tissue. We placed the tissue in 9 mL of ice-cold 0.9% saline solution and homogenized it, followed by centrifugation at 3500× *g* for 15 min at 4 °C. Supernatants were collected for antioxidant analysis. The levels of total antioxidant capacity (T-AOC), glutathione peroxidase (GSH-Px), total superoxide dismutase (T-SOD), catalase (CAT), hydrogen peroxide (H_2_O_2_), and malondialdehyde (MDA) in plasma, liver, and intestinal tissues were determined using commercial kits (Nanjing Jiancheng Bioengineering Institute, Nanjing, China).

### 2.7. Serum Diamine Oxidase

Serum DAO concentration was determined using an ultraviolet colorimetry commercial kit (Nanjing Jiancheng Bioengineering Institute, Nanjing, China), following the manufacturer’s instructions.

### 2.8. Intestinal Morphology

Intestinal segments (duodenum, jejunum, ileum; ≈1 cm; mid-segment) were fixed in 4% paraformaldehyde for 72 h, dehydrated, cleared, embedded in paraffin, and sectioned at 4 μm thickness using the Leica RM2255 Automated Microtome (Leica Biosystems GmbH, Nussloch, Germany). Sections were stained with hematoxylin and eosin (Thermo Fisher Scientific Inc., Waltham, MA, USA). Villus height (VH; from tip to villus–crypt junction), crypt depth (CD; from villus–crypt junction to base), villus width (VW; measured at the middle of the villi), and villus area (VA; OLYMPSBBX-41TF measurement provides data) were measured according to a previous study [15]. We performed using an Olympus BX-41TF microscope (Olympus, Tokyo, Japan). For each section, 10 intact villi (full length, absence of folding) were randomly selected, and VH, CD, VW, and VA were measured using Olympus cellSens Standard software version 3.2 (OLYMPSBBX-41TF, Olympus Corporation, Tokyo, Japan). The experiment was performed by a trained person who was blinded to the groups. The VH/CD ratio was calculated.

### 2.9. Transcription Level of Genes in the Liver and Intestine

Approximately 0.1 g of liver or intestinal tissue was placed in a 2 mL RNase-free tube, and 1 mL of Trizol reagent (Takara, Dalian, China) was added. Samples were homogenized at 4 °C, and total RNA was extracted as described by Hou et al. [16]. RNA purity (A260:A280 = 1.8–2.0) and concentration were determined using a NanoDrop^®^ ND2000 spectrophotometer (Thermo Scientific, Wilmington, DE, USA). Reverse transcription was performed on 1 µg of total RNA using the HiScript III RT SuperMix kit (Takara, Dalian, China) according to the manufacturer’s instructions. Real-time quantitative PCR (qPCR) was performed using an ABI 7500 system (Applied Biosystems, Foster City, CA, USA) and Taq Pro Universal SYBR qPCR Master Mix (Takara, Dalian, China). The 10 μL reaction mixture contained 5 μL Master Mix, 1 μL cDNA (50 ng), 3.6 μL RNase-free water, and 0.2 μL of each primer (10 μmol/L). The program was: pre-denaturation at 95 °C for 30 s; 40 cycles of 95 °C for 5 s and 60 °C for 34 s. Melting curve analysis was performed at 95 °C for 15 s, 60 °C for 1 min, and 95 °C for 15 s. Gene expression levels were quantified using the 2^−ΔΔCt^ method [17,18], with β-actin as the reference gene. Primer sequences are provided in Table 2.

### 2.10. Statistical Analysis

The experimental data were analyzed using Statistical Package for the Social Sciences (SPSS) version 27.0 (SPSS, Inc., Chicago, IL, USA) statistical software. Data distribution and homogeneity of variances was verified using the Shapiro–Wilk and Levene’s tests, respectively. All the data had an appropriate normal distribution and homogeneity of variances. Growth performance data were analyzed using independent t-tests. Other data were analyzed using a two-way ANOVA with a 2 × 2 factorial design to assess the effects of LPS challenge and NG supplementation on outcomes, as well as their interaction. When interactions were observed, further analysis was conducted using one-way ANOVA followed by Duncan’s multiple comparison test. Differences were considered statistically significant at *p* < 0.05.

## 3. Results

### 3.1. Growth Performance

As shown in Table 3, dietary supplementation with NG had no significant effect on BW, ADG, ADFI, or FCR of broilers during days 1–21, 22–35, or across the whole experimental period (*p* > 0.05).

### 3.2. Plasma Biochemical Parameters

According to Table 4, LPS challenge markedly increased plasma ALT and ALP activities, while reducing TP, ALB, TB, and GGT concentrations (*p* < 0.05). NG supplementation significantly increased plasma HDL levels (*p* < 0.05). Moreover, significant interactions between dietary NG and LPS injection were observed for TB, ALT, ALP, GGT, TG, TC, LDL, and LDH (*p* < 0.05). Specifically, NG supplementation reduced plasma ALT, ALP, and LDH, while increasing TB, TG, TC, and LDL levels in LPS-challenged broilers (*p* < 0.05).

### 3.3. Organ Index

As shown in Table 5, dietary supplementation with NG had no significant effect on organ index.

### 3.4. Antioxidant Status

As shown in Table 6, LPS challenge significantly increased CAT and SOD activities but reduced plasma MDA concentrations (*p* < 0.05). Dietary NG alone significantly decreased plasma CAT activity (*p* < 0.05). A significant NG × LPS interaction was observed for plasma MDA (*p* < 0.05), with NG elevating MDA in unstimulated broilers (*p* < 0.05). In the liver, LPS substantially increased T-AOC and T-SOD activities while decreasing MDA (*p* < 0.05). NG × LPS interaction was also detected for hepatic GSH-Px activity (*p* < 0.05), with NG enhancing GSH-Px activity in unstimulated broilers (*p* < 0.05).

As shown in Table 7, LPS reduced CAT activity in the duodenum, whereas NG supplementation significantly increased CAT activity and reduced MDA levels (*p* < 0.05). In the jejunum, LPS significantly increased CAT activity and H_2_O_2_ concentrations (*p* < 0.05), while NG supplementation reduced both H_2_O_2_ and MDA (*p* < 0.05). A significant NG × LPS interaction was observed for jejunal T-SOD activity (*p* < 0.05), with NG increasing T-SOD in LPS-challenged broilers. In the ileum, LPS significantly increased H_2_O_2_ and MDA, while NG increased CAT and GSH-Px activities and reduced H_2_O_2_ and MDA (*p* < 0.05). Interactive effects of NG × LPS were found on ileal T-AOC, T-SOD, and GSH-Px (*p* < 0.05). Without LPS challenge, NG reduced ileal T-AOC and T-SOD (*p* < 0.05), whereas in LPS-challenged broilers, NG supplementation restored these indices.

### 3.5. Intestinal Barrier Function

**Figure 1:**
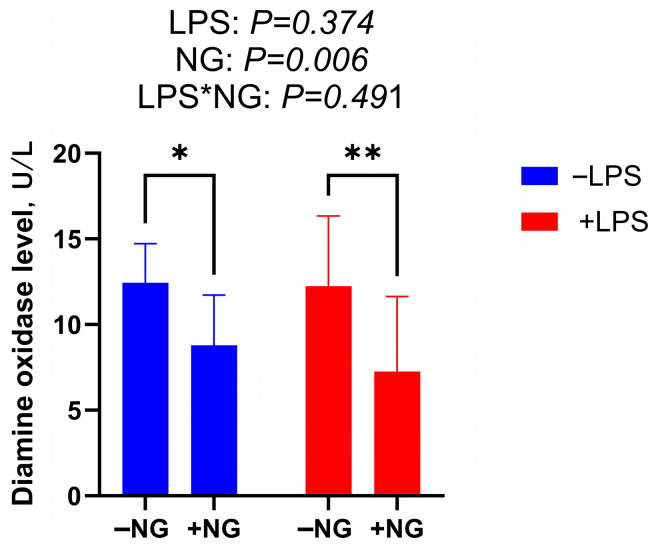
The effect of NG on serum DAO contents in broilers challenged with LPS. * indicates a significant difference (*p* < 0.05), ** indicates a highly significant difference (*p* < 0.01). Data were presented with the means and SEM (n = 12). −LPS, injected with saline; +LPS, injected with LPS; −NG, fed basal diets; +NG, fed basal diets supplemented with 200 mg/kg NG.

As shown in Figure 1, NG supplementation significantly decreased serum DAO concentration (*p* < 0.05).

**Figure 2:**
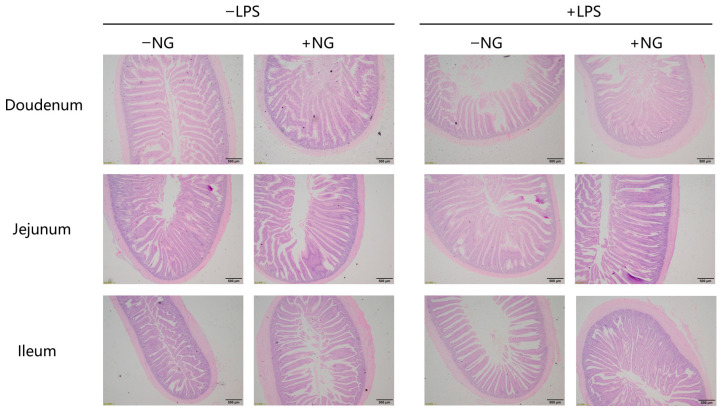
The images of intestinal morphology. −LPS, injected with saline; +LPS, injected with LPS; −NG, fed basal diets; +NG, fed basal diets supplemented with 200 mg/kg NG. Each image is magnified 500×.

The images of intestinal morphology are presented in Figure 2. According to Table 8, NG supplementation significantly increased VH and VH/CD in the duodenum and jejunum (*p* < 0.05). A significant NG × LPS interaction was detected for duodenal CD and VH/CD (*p* < 0.05). In particular, NG improved VH/CD in LPS-challenged broilers.

As shown in Table 9, LPS significantly upregulated *Claudin-1* expression in the jejunum and downregulated *ZO-1* and *Occludin* expression in the duodenum (*p* < 0.05). NG supplementation upregulated *ZO-1* and *Occludin* expression in the duodenum while downregulating *Mucin-2* in the duodenum and ileum (*p* < 0.05). NG × LPS interactions were observed for jejunal *Mucin-2* and ileal *ZO-1* expression (*p* < 0.05). In LPS-challenged broilers, NG upregulated ileal *ZO-1* and downregulated ileal *Mucin-2* expression (*p* < 0.05).

### 3.6. Immune Response

As shown in Table 10, LPS challenge significantly upregulated *IL-1β*, *IL-8*, *iNOS*, and *TNF-α* expression in the liver (*p* < 0.05). NG supplementation upregulated *NF-κB* expression (*p* < 0.05). NG × LPS interactions were observed for *TLR4* and *IFN-γ* expression (*p* < 0.05). Without LPS challenge, NG increased *TLR4* and *IFN-γ* expression, whereas in LPS-challenged broilers, NG reversed their expression.

As shown in Table 11, LPS upregulated *IL-1β*, *IL-8*, and *IFN-γ* in the duodenum; *IL-1β*, *IL-8*, *TNF-α*, and *IFN-γ* in the jejunum; and *IL-1β*, *IL-8*, *TNF-α*, *NF-κB*, and *IFN-γ* in the ileum (*p* < 0.05), while downregulating *TLR4* in the duodenum. NG significantly downregulated *IFN-γ* in the jejunum (*p* < 0.05). An NG × LPS interaction was observed for jejunal *TNF-α* expression (*p* < 0.05), with NG reducing *TNF-α* expression under LPS challenge.

As shown in Table 12, LPS significantly increased *MMP-9* and *XIAP* expression while decreasing *MMP-13* and *Bcl-2* expression in the liver (*p* < 0.05). NG supplementation increased *XIAP* and *Bcl-2* expression (*p* < 0.05). NG × LPS interactions were observed for *MMP-13* and *Bcl-2* expression (*p* < 0.05). Specifically, under LPS challenge, NG further reduced hepatic *MMP-13* expression.

## 4. Discussion

During broiler production, pathogenic infections, feed-derived toxins, and environmental stressors frequently induce immune stress, ultimately impairing growth performance and reducing economic efficiency [19]. LPS is commonly used to model immune stress due to its reproducibility, controllability, and well-characterized pro-inflammatory mechanisms [20]. Upon entering the host cell, LPS activates signaling cascades that drive nuclear translocation of NF-κB, resulting in the release of pro-inflammatory cytokines such as TNF-α, IL-6, and IL-1β. These cytokines exacerbate lipid metabolism disorders and tissue damage [11,12]. However, the limitations of the LPS model must be acknowledged: as an acute endotoxin challenge, it primarily mimics the acute phase of immune stress induced by Gram-negative bacterial infections and may not fully replicate the complexity, low intensity, and persistent nature of natural chronic immune stress observed in commercial broiler flocks. Natural chronic immune stress typically arises from prolonged cumulative exposure to multiple stressors (e.g., pathogens, mycotoxins, poor air quality, nutritional imbalances), leading to sustained immune activation rather than the rapid transient inflammatory response triggered by a single LPS injection. Therefore, caution is warranted when extrapolating the NG protective effects observed in this acute model to real-world production scenarios. Future studies employing chronic immune stress models—such as repeated low-dose LPS administration or mixed pathogen challenges—will be essential for validating NG’s long-term efficacy. Naringin (NG), a major bioactive flavonoid in citrus fruits, exhibits multiple biological activities. Pravin B. et al. [21] evaluated the antioxidant capacity of NG using DPPH and radical scavenging assays, demonstrating that NG possesses an antioxidant effect that reduces oxidative stress. Li Z.H. et al. [22] demonstrated that NG significantly ameliorates acetaminophen (APAP)-induced liver injury in mice in a dose-dependent manner and reduces the expression levels of liver injury markers. NG can inhibit the progression of cancers in various sites, serving as an effective alternative therapy to alleviate symptoms in cancer patients. Its anticancer effects exhibit multifaceted properties, regulating multiple cellular signaling pathways, suppressing the production of cytokines and growth factors, and arresting the cell cycle [23]. Pari, L. et al. [24] demonstrated that NG mitigated nickel toxicity in rat livers by reversing the activity of hepatic marker enzymes, reducing levels of lipid peroxidation markers, enhancing antioxidant cascades, and lowering nickel concentrations in the liver. NG improved glucose and lipid metabolism as well as endothelial dysfunction in rats with type 2 diabetes by downregulating oxidative stress and inflammatory responses [25]. However, The effects of NG on broiler growth performance and its role in regulating the immune stress response during LPS challenge in broilers have been rarely reported, necessitating further investigation.

Previous reports demonstrated that NG improved growth and bone health in broilers with tibial dysplasia [26]. In contrast, our study showed that 200 mg/kg NG supplementation did not significantly influence growth performance across different stages. This discrepancy may be related to variations in basal diet composition, feeding duration, or bioactive compound content.

Biochemical parameters are sensitive indicators of organ function and metabolic state. In this study, LPS increased plasma ALT and LDH activities, consistent with liver injury, whereas NG supplementation mitigated these effects. This agrees with Ahmed et al. [27], who reported that NG alleviated chemically induced hepatotoxicity. Furthermore, LPS reduced TB, an endogenous antioxidant, while NG supplementation restored TB levels, suggesting enhanced antioxidative defense [28]. LPS also elevated ALP activity, indicating impaired digestive and metabolic regulation [29]. NG attenuated this response, further supporting its hepatoprotective role. Lipid metabolism was markedly altered by LPS, which decreased plasma TG, TC, and LDL. Notably, NG supplementation reversed these changes, highlighting its role in maintaining lipid transport and circulation homeostasis.

In the internal environment of organisms, the T-AOC serves as a comprehensive indicator reflecting the antioxidant potential of all antioxidant substances and enzymes in animals [30]. Both T-SOD and GSH-Px act as critical scavengers of oxygen-derived free radicals, effectively mitigating oxidative stress in the body [31]. The interaction between free radicals and lipid molecules induces lipid peroxidation, with MDA being a key terminal product of this process [32]. CAT is a marker enzyme of peroxisomes, and it plays a pivotal role in the decomposition of H_2_O_2_ [33]. In vitro experiments have shown that NG significantly ameliorates the reduction in T-SOD, CAT, and GSH-Px activities induced by hypoxia/reoxygenation in H9C2 cells. Additionally, NG markedly decreases intracellular reactive oxygen species (ROS) and MDA levels, thereby reducing oxidative stress damage [34]. The results of this experimental study indicate that under LPS challenge, the addition of naringin reduced plasma MDA levels and decreased hepatic GSH-Px activity. In the duodenum, NG supplementation alleviated the LPS-induced decrease in CAT activity and reduced MDA levels, indicating that NG has the potential to improve antioxidant defense in this region. In the jejunum, under LPS challenge, NG also significantly enhanced T-SOD activity, further supporting its role in modulating oxidative stress responses in the gastrointestinal tract. In the ileum, under LPS challenge, NG enhanced CAT and GSH-Px activities. Collectively, these results suggest that NG may form an antioxidant defense barrier in the intestine by activating the glutathione system and enhancing the H_2_O_2_-scavenging capacity mediated by CAT.

DAO is an intracellular enzyme predominantly found in the villus cells of the mammalian intestine. When the intestinal mucosa is compromised by external factors, the rupture of villus cells leads to the release of DAO into the bloodstream, causing elevated DAO levels. These elevated levels serve as a biomarker for the extent of intestinal mucosal damage [35]. In the present study, NG supplementation significantly reduced serum DAO levels in broilers, suggesting that NG enhances intestinal integrity and mitigates intestinal injury. Zhang et al. [36] reported that under LPS-induced stress conditions, broilers exhibited significantly decreased VH and VH/CD in the jejunum. In line with these findings, our study demonstrated that NG supplementation markedly enhanced the duodenal CD and VH/CD ratio under LPS challenge. These results collectively indicate that NG effectively ameliorates inflammation-induced villus atrophy and crypt hyperplasia, thereby contributing to the preservation of intestinal morphology and function. It should be noted that intestinal morphometry may exhibit technical variability, including sampling site selection and villus selection criteria. To minimize this variability, we established a standardized sampling protocol. Despite these measures, the inherent biological variability of intestinal structure cannot be entirely eliminated. Furthermore, intestinal morphology is closely associated with microbial community composition. Future studies should integrate 16S rRNA sequencing to explore associations between NG-induced morphological improvements and microbial community shifts, while employing nutrient absorption assays (e.g., D-xylose absorption tests) to directly validate functional outcomes.

The intestinal barrier consists of mechanical, immune, chemical, and microbial components. Tight junctions form junctional complexes that, together with epithelial cells, constitute the mechanical barrier [37]. Claudin-1 maintains and regulates cellular connections and intestinal defense, while occludin reduces permeability across cell membranes, effectively filtering both small and large molecules. ZO-1 is located on the cytoplasmic side of the cell membrane and links the tight junctions to the actin cytoskeleton [38]. Mucin, primarily secreted by goblet cells, covers the surface of intestinal mucosal cells, playing a key role in isolating harmful substances within the intestinal tract. Mucin-2, one of the most abundant mucins secreted by goblet cells, interacts dynamically with intestinal epithelial cells, the microbiota, and the host immune system to maintain intestinal mucosal homeostasis [39]. Cao et al. [40] demonstrated that NG could enhance the expression of ZO-1 and occludin in the colon of mice with DSS-induced ulcerative colitis. In alignment with their findings, our study revealed that under LPS challenge, NG supplementation markedly increased ZO-1 expression in the ileum and reduced Mucin-2 expression in the jejunum. The ileum-specific upregulation of ZO-1 and jejunum-specific suppression of Mucin-2 by NG may counteract LPS-induced oxidative stress via the Nrf2/KEAP1 pathway, and inhibit IFN-γ-driven mucus hypersecretion through suppression of the TLR4/IRF3 pathway—though this interpretation is speculative. It is based on the known roles of these pathways in oxidative stress and mucus regulation [41,42]. The coordinated upregulation of duodenal ZO-1 and downregulation of Mucin-2 suggest that NG promotes a ‘tight-junction-dominant’ barrier phenotype while inhibiting pathological mucus hyperplasia.

Alterations in the intestinal immune system can lead to changes in the intestinal mechanical barrier, with the homeostasis of this immune system playing a crucial role in both intestinal and systemic health [43]. Jiang et al. [44] reported that LPS challenge significantly upregulated the relative expression of IL-1β, IL-6, and TNF-α genes in the duodenum of broilers. In the present study, LPS stimulation notably increased the relative expression of IL-1β, IL-8, iNOS, and TNF-α in the liver; IL-1β, IL-8, and IFN-γ in the duodenum; IL-1β, IL-8, TNF-α, and IFN-γ in the jejunum; and IL-1β, IL-8, TNF-α, NF-κB, and IFN-γ in the ileum, while significantly downregulating duodenal TLR4 expression. These findings align with previous studies. Bi et al. [45] demonstrated that NG significantly downregulated the expression of IL-1, IL-6, TNF-α, and other inflammation-related proteins in LPS-induced human inflammatory vein endothelial cells. In the current study, NG effectively mitigated the LPS-induced upregulation of TNF-α expression in the jejunum, which is consistent with their core findings. NG also maintained baseline TLR4 expression levels, suggesting its potential to stabilize the TLR4-MD2 complex conformation or inhibit endocytic degradation pathways, thereby preserving innate immune recognition function. In jejunal tissue, NG significantly suppressed the overexpression of TNF-α and IFN-γ, consistent with established mechanisms by which flavonoids inhibit NF-κB nuclear translocation. Collectively, these results demonstrate that LPS stimulation drives pro-inflammatory gene expression, while NG counteracts the detrimental effects of LPS by attenuating such pro-inflammatory responses.

LPS is an endotoxin composed of lipids and polysaccharides that triggers excessive production of inflammatory factors, leading to immune stress and subsequent liver injury in animals [46]. The MMP-9 gene, a member of the matrix metalloproteinase (MMP) family, primarily functions in degrading collagen and other components of the extracellular matrix, facilitating tissue remodeling and cell migration [47]. MMP-13, another MMP family member, plays a key role in degrading cartilage matrix during skeletal development and remodeling, promoting cartilage extracellular matrix restructuring [48]. XIAP is the most potent endogenous inhibitor of apoptosis, while BCL-2 is an anti-apoptotic protein that mainly regulates the mitochondrial apoptosis pathway [49]. In the present study, LPS challenge significantly increased the relative expression of hepatic MMP-9, confirming LPS-induced liver injury in broilers. In contrast, LPS challenge downregulated hepatic MMP-13 expression. This suppression may reflect a dominance of acute-phase inflammation, as MMP-13-mediated collagen remodeling typically activates in later stages of tissue repair. Dietary NG supplementation significantly upregulated the relative expression of XIAP and BCL-2 in the livers of broilers, indicating NG’s anti-apoptotic protective effect against hepatic damage. These findings are consistent with NG’s ability to reduce plasma ALT levels under LPS challenge.

A key limitation of this study is the lack of protein-level confirmation for the observed gene expression changes. Although we detected consistent mRNA expression patterns for key genes involved in inflammation (e.g., TNF-α), barrier function (e.g., ZO-1), and apoptosis (e.g., BCL-2, XIAP), mRNA abundance does not always correlate with functional protein levels due to post-transcriptional regulation and post-translational modifications. Therefore, Western blotting or immunohistochemistry of these proteins would provide more direct evidence for NG’s regulatory role in these pathways. Furthermore, future work should integrate multi-omics approaches such as proteomics and metabolomics to bridge the gap between transcriptional responses and actual biological activity, and to identify novel molecular targets for NG in mitigating LPS-induced immune stress.

## 5. Conclusions

Dietary supplementation with NG alleviated LPS-induced liver and intestinal injury in broilers. NG modulated plasma biochemical markers, improved intestinal morphology, enhanced antioxidant enzyme activity, regulated tight junction protein expression, and reduced pro-inflammatory cytokine expression. Collectively, these findings suggest NG is a promising natural feed additive for protecting broilers against immune stress and enhancing intestinal health.

## Figures and Tables

**Table 1:** Ingredients and nutrient composition of basal diets.

Items (%, Unless Otherwise Indicated)	1–21 Days of Age	22–35 Days of Age
Corn	51.73	57.68
Soybean meal (crude protein, 43.0%)	40.73	35.15
Soybean oil	3.36	3.66
Dicalcium phosphate	1.92	1.33
Limestone	1.16	1.26
Sodium chloride	0.35	0.35
DL-Methionine	0.26	0.13
Choline chloride (50%)	0.25	0.20
Multi-Minerals ^1^	0.20	0.20
Multi-Vitamins ^2^	0.04	0.04
Calculated nutrient levels		
Crude protein	24.14	21.95
ME (MJ/kg)	12.22	12.56
Ca	1.15	0.99
Available P	0.62	0.48
Lysine	1.39	1.23
Methionine	0.60	0.45
Methionine + Cystine	0.96	0.79
Threonine	0.96	0.87
Tryptophan	0.27	0.24

^1^ The Multi-Minerals supplied the following per kilogram of diet: copper, 8 mg; iron, 80 mg; zinc, 75 mg; manganese, 100 mg; selenium, 0.15 mg; iodine, 0.35 mg. ^2^ The Multi-Vitamins supplied the following per kilogram of diet: vitamin A, 8000 IU; vitamin D, 3000 IU; vitamin E, 44 IU; vitamin K, 3.2 mg; vitamin B_1_, 3.0 mg; vitamin B_2_, 4.0 mg; vitamin B_12_, 0.025 mg; biotin, 0.0325 mg; folic acid, 2.00 mg; nicotinic acid, 15 mg; pantothenic acid, 15 mg.

**Table 2:** The primer sequences list ^1^.

Gene Name	Primer Sequence (5′ to 3′)	NCBI Number
*β-Actin*	F:GAGAAATTGTGCGTGACATCA	NM-205518.1
	R:CCTGAACTCATTGCCA	
*Claudin-1*	F:CATACTCCTGGGTCTGGTTGGT	NM-001013611.2
	R:GACAGCCATCCGCATCTTCT	
*ZO-1*	F: CTTCAGGTGTTTCTCTTCCTCCTC	XM-040680632.1
	R:CTGTGGTTTCATGGCTGGATC	
*Occludin*	F:ACGGCAGCACCTACCTCAA	NM-205128.1
	R:GGGCGAAGAAGCAGATGAG	
*Mucin2*	F:TTCATGATGCCTGCTCTTGTG	NM-001318434.1
	R:CCTGAGCCTTGGTACATTCTTGT	
*IFN-γ*	F:AGCTGACGGTGGACCTATTATT	NM-205149.1
	R:GGCTTTGCGCTGGATTC	
*TLR4*	F:AGTCTGAAATTGCTGAGCTCAAAT	NM-001030693.1
	R:GCGACGTTAAGCCATGGAAG	
*IL-1β*	F:ACTGGGCATCAAGGGCTA	NM-204524.1
	R:GGTAGAAGATGAAGCGGGTC	
*IL-8*	F:CGCTGTCACCGCTTCTTCA	NM-205498.1
	R:CGTCTCCTTGATCTGCTTGATG	
*iNOS*	F:CCTGTACTGAAGGTGGCTATTGG	NM-204961.1
	R:AGGCCTGTGAGAGTGTGCAA	
*NF-κB*	F:GTGTGAAGAAACGGGAACTG	NM-205129.1
	R:GGCACGGTTGTCATAGATGG	
*TNF-α*	F:CTGTGGGGCGTGCAGTG	XM-040647309.1
	R:ATGAAGGTGGTGCAGATGGG	
*MMP-13*	F:TGGAACACTCCAGAGACCCT	NM-002427.4
	R:CTCTGTCTCCAGCACCATAGA	
*MMP-9*	F:ATAGATGATGCCTTCCGGCG	XM-06334928.1
	R:CCATCACCATGCTCTTGGCT	
*BCL-2*	F:GCTGCTTTACTCTTGGGGGT	NM-205339.2
	R:CTTCAGCACTATCTCGCGGT	
*XIAP*	F:AACCTGGTGATCGAGCTTGG	XM-063346332.1
	R:GTCCCGACCCAGGACAAAAA	
*Caspase-3*	F:TGGTGGAGGTGGAGGAGC	NM-204725.1
	R:TGTCTGTCATCATGGCTCTTG	

^1^ The primers were designed and synthesized by Shanghai Sangon Bioengineering Co., Ltd., Shanghai, China. *Claudin-1*, *Occludin*, and *ZO-1* were tight junction proteins; *IFN-γ*, Interferon gamma; *TLR4*, Toll-like receptor 4; *IL-1β*, Interleukin-1β; *IL-8*, Interleukin-8; *iNOS*, Inducible nitric oxide synthase; *NF-κB*, Nuclear factor kappa-light-chain-enhancer of activated B cells; *TNF-α*, Tumor necrosis factor alpha; *MMP-13*, Matrix metalloproteinase-13; *MMP-9*, Matrix metalloproteinase-9; *BCL-2*, B-cell lymphoma 2; *XIAP*, X-linked inhibitor of apoptosis protein.

**Table 3:** The effect of NG on the growth performance of broilers ^1^.

Items	CON	NG	*p*-Value
Day 1 BW (g)	47.06 ± 0.91	46.72 ± 0.55	0.879
Day 21 BW (g)	949.28 ± 48.84	971.84 ± 41.50	0.614
Day 35 BW (g)	1849.31 ± 53.10	1884.82 ± 85.69	0.315
Days 1 to 21			
ADG (g)	42.95 ± 2.24	44.02 ± 1.82	0.599
ADFI (g)	57.25 ± 1.58	59.48 ± 1.92	0.242
FCR	1.33 ± 0.07	1.35 ± 0.06	0.893
Days 22 to 35			
ADG (g)	64.28 ± 3.43	65.21 ± 5.30	0.142
ADFI (g)	117.72 ± 5.84	119.93 ± 10.12	0.817
FCR	1.83 ± 0.11	1.84 ± 0.12	0.617
Days 1 to 35			
ADG (g)	51.46 ± 0.92	52.52 ± 2.21	0.355
ADFI (g)	81.44 ± 2.56	83.66 ± 4.79	0.909
FCR	1.58 ± 0.04	1.59 ± 0.06	0.467

^1^ Data were presented with the means ± SD (n = 6). CON, control; NG, naringin; BW, average body weight; ADG, average daily gain; ADFI, average daily feed intake; FCR, feed conversion ratio.

**Table 4:** The effect of NG on plasma biochemical parameters of broilers challenged with LPS ^1^.

Items	−LPS		+LPS		SEM	*p*-Value		
	−NG	+NG	−NG	+NG		LPS	NG	LPS × NG
TP (g/L)	30.99	28.97	28.04	27.91	0.499	0.043	0.272	0.330
ALB (g/L)	12.92	12.76	11.95	11.47	0.177	0.001	0.330	0.617
GLB (g/L)	18.07	16.21	16.09	16.44	0.387	0.259	0.328	0.154
TB (μmol/L)	10.05 ^a^	8.77 ^ab^	6.38 ^c^	8.39 ^b^	0.292	<0.001	0.424	0.001
ALT (U/L)	2.41 ^b^	2.17 ^b^	3.09 ^a^	2.18 ^b^	0.118	0.031	0.720	<0.001
AST (U/L)	226.00	219.67	223.00	214.33	3.211	0.077	0.216	0.125
ALP (U/L)	3474.61 ^b^	3324.60 ^b^	4464.18 ^a^	2897.94 ^b^	159.281	0.321	0.004	0.015
GGT (U/L)	31.67 ^a^	19.83 ^c^	24.64 ^b^	23.67 ^b^	0.949	0.044	0.398	<0.001
TG (mmol/L)	0.47 ^b^	0.38 ^c^	0.33 ^c^	0.56 ^a^	0.017	0.445	0.005	<0.001
TC (mmol/L)	3.53 ^a^	3.28 ^a^	2.84 ^b^	3.47 ^a^	0.084	0.108	0.226	0.006
HDL (mmol/L)	2.09	2.28	1.93	2.21	0.044	0.268	0.025	0.615
LDL (mmol/L)	0.36 ^b^	0.35 ^b^	0.29 ^b^	0.53 ^a^	0.016	0.073	<0.001	<0.001
LDH (U/L)	596.99 ^b^	610.99 ^b^	728.71 ^a^	590.48 ^b^	12.893	0.112	0.077	0.032

^a–c^ Means labeled with no common superscript in the same row differed significantly (*p* < 0.05). ^1^ Data were presented with the means and SEM (n = 12). −LPS, injected with saline; +LPS, injected with LPS; −NG, fed basal diets; +NG, fed basal diets supplemented with 200 mg/kg NG. TP, Total protein; ALB, albumin; GLB, globulin; TB, total bilirubin; AST, aspartate aminotransferase; ALT, alanine aminotransferase; ALP, alkaline phosphatase; GGT, glutamyl transpeptidase; TG, triglycerides; TC, total cholesterol; HDL, high-density lipoprotein; LDL, low-density lipoprotein; LDH, lactate dehydrogenase.

**Table 5:** The effect of NG on organ index in broilers challenged with LPS ^1^.

Items	−LPS		+LPS		SEM	*p*-Value		
	−NG	+NG	−NG	+NG		LPS	NG	LPS × NG
Thymus (g/kg)	1.80	1.42	1.59	1.60	0.057	0.916	0.092	0.079
Spleen (g/kg)	1.03	0.80	0.96	0.92	0.032	0.699	0.069	0.114
Bursa of Fabricius (g/kg)	2.28	2.61	2.55	2.44	0.081	0.756	0.502	0.175
Liver (g/kg)	20.72	20.40	22.64	22.55	0.271	<0.001	0.662	0.816

^1^ Data were presented with the means and SEM (n = 12). −LPS, injected with saline; +LPS, injected with LPS; −NG, fed basal diets; +NG, fed basal diets supplemented with 200 mg/kg NG.

**Table 6:** The effect of NG on plasma and hepatic antioxidant status in broilers challenged with LPS ^1^.

Items	−LPS		+LPS		SEM	*p*-Value		
	−NG	+NG	−NG	+NG		LPS	NG	LPS × NG
Plasma								
T-AOC (mM)	1.76	1.77	1.84	1.78	0.013	0.069	0.257	0.143
CAT (U/mL)	8.99	2.53	18.17	12.49	0.978	<0.001	<0.001	0.614
GSH-Px (U/mL)	1427.53	1450.81	1495.29	1434.56	31.993	0.699	0.779	0.530
T-SOD (U/mL)	51.21	55.94	84.08	73.84	3.470	<0.001	0.640	0.208
H_2_O_2_ (mmol/L)	34.96	43.89	34.05	34.84	1.640	0.123	0.133	0.205
MDA (nmol/mL)	1.57 ^b^	4.18 ^a^	1.94 ^b^	1.73 ^b^	0.188	<0.001	<0.001	<0.001
Liver								
T-AOC (mmol/gprot)	1.28	1.25	1.43	1.40	0.017	<0.001	0.134	0.859
CAT (U/mgprot)	32.70	41.03	36.82	44.08	1.996	0.364	0.053	0.891
GSH-Px (U/mgprot)	424.25 ^c^	530.89 ^b^	644.54 ^a^	561.73 ^ab^	19.992	<0.001	0.715	0.006
T-SOD (U/mgprot)	285.91	298.34	336.44	372.18	9.317	<0.001	0.129	0.456
H_2_O_2_ (mmol/gprot)	58.87	53.07	51.58	53.97	1.478	0.286	0.567	0.173
MDA (nmol/mgprot)	4.28	4.70	2.46	3.69	0.262	0.004	0.082	0.384

^a–c^ Means labeled with no common superscript in the same row differed significantly (*p* < 0.05). ^1^ Data were presented with the means and SEM (n = 12). −LPS, injected with saline; +LPS, injected with LPS; −NG, fed basal diets; +NG, fed basal diets supplemented with 200 mg/kg NG. T-AOC, total antioxidant capacity; GSH-Px, glutathione peroxidase; T-SOD, total superoxide dismutase; CAT, catalase; H_2_O_2_, hydrogen peroxide and MDA, malondialdehyde.

**Table 7:** The effect of NG on intestinal antioxidant status in broilers challenged with LPS ^1^.

Items	−LPS		+LPS		SEM	*p*-Value		
	−NG	+NG	−NG	+NG		LPS	NG	LPS × NG
Duodenum								
T-AOC (mmol/gprot)	2.24	2.19	2.17	2.22	0.039	0.823	0.965	0.515
CAT (U/mgprot)	10.40	27.41	6.58	17.49	1.533	<0.001	<0.001	0.053
GSH-Px (U/mgprot)	194.85	219.57	227.89	137.78	21.334	0.578	0.456	0.195
T-SOD (U/mgprot)	154.71	173.15	172.89	159.83	4.718	0.798	0.777	0.104
H_2_O_2_ (mmol/gprot)	20.06	18.85	25.14	20.60	0.911	0.054	0.103	0.338
MDA (nmol/mgprot)	2.33	1.13	2.80	1.99	0.206	0.081	0.011	0.612
Jejunum								
T-AOC (mmol/gprot)	0.70	0.69	0.69	0.71	0.009	0.702	0.908	0.293
CAT (U/mgprot)	3.84	3.97	4.99	5.61	0.199	<0.01	0.281	0.483
GSH-Px (U/mgprot)	238.48	192.44	224.75	263.48	13.000	0.273	0.888	0.108
T-SOD (U/mgprot)	94.93 ^a^	96.86 ^a^	76.95 ^b^	92.13 ^a^	4.286	0.064	0.005	0.002
H_2_O_2_ (mmol/gprot)	27.62	21.29	32.60	25.24	1.205	0.046	0.003	0.813
MDA (nmol/mgprot)	3.73	1.78	3.63	1.73	0.230	0.299	0.001	0.202
Ileum								
T-AOC (mmol/gprot)	2.34 ^a^	1.80 ^b^	1.81 ^b^	1.91 ^b^	0.045	0.001	0.001	<0.001
CAT (U/mgprot)	3.26	10.34	3.17	9.22	0.703	0.424	<0.001	0.494
GSH-Px (U/mgprot)	84.50 ^a^	84.83 ^a^	60.29 ^c^	72.41 ^bc^	2.045	<0.001	0.022	0.029
T-SOD (U/mgprot)	62.21 ^a^	48.41 ^b^	52.39 ^b^	54.83 ^b^	1.429	0.495	0.027	0.002
H_2_O_2_ (mmol/gprot)	7.80	4.60	10.46	8.72	0.638	0.004	0.031	0.513
MDA (nmol/mgprot)	8.83 ^a^	4.60 ^b^	9.49 ^a^	9.33 ^a^	0.581	0.011	0.036	0.051

^a–c^ Means labeled with no common superscript in the same row differed significantly (*p* < 0.05). ^1^ Data were presented with the means and SEM (n = 12). −LPS, injected with saline; +LPS, injected with LPS; −NG, fed basal diets; +NG, fed basal diets supplemented with 200 mg/kg NG. T-AOC, total antioxidant capacity; GSH-Px, glutathione peroxidase; T-SOD, total superoxide dismutase; CAT, catalase; H_2_O_2_, hydrogen peroxide and MDA, malondialdehyde.

**Table 8:** The effect of NG on the intestinal morphology of broiler challenged with LPS ^1^.

Items	−LPS		+LPS		SEM	*p*-Value		
	−NG	+NG	−NG	+NG		LPS	NG	LPS × NG
Duodenum								
VH (μm)	1094.23	1206.62	1058.86	1323.60	31.797	0.425	0.001	0.145
CD (μm)	137.06 ^b^	179.73 ^a^	153.06 ^ab^	129.98 ^b^	5.982	0.081	0.298	0.002
VW (μm)	157.06	138.13	146.44	167.19	5.987	0.449	0.94	0.113
VA (μm^2^)	163,849.27	177,766.26	156,094.95	181,832.87	6206.423	0.885	0.132	0.644
VH/CD	8.32 ^b^	6.90 ^b^	7.16 ^b^	10.29 ^a^	0.383	0.059	0.142	0.001
Jejunum								
VH (μm)	1060.68	1367.86	1083.80	1325.52	43.367	0.890	0.001	0.637
CD (μm)	146.99	146.68	175.69	143.86	5.881	0.261	0.167	0.175
VW (μm)	126.93	126.10	148.01	125.42	3.613	0.129	0.084	0.107
VA (μm^2^)	142,396.87	175,103.10	149,210.97	169,502.83	7390.928	0.967	0.086	0.677
VH/CD	7.37	9.41	6.27	9.48	0.400	0.395	<0.001	0.332
Ileum								
VH (μm)	915.37	1260.56	808.94	1051.03	88.045	0.374	0.108	0.770
CD (μm)	148.00	126.56	137.18	132.04	4.488	0.770	0.157	0.377
VW (μm)	131.25	137.89	131.15	115.18	4.834	0.252	0.634	0.256
VA (μm^2^)	117,795.08	121,670.77	134,290.00	110,539.00	8794.181	0.887	0.601	0.468
VH/CD	6.21	10.45	5.94	7.91	0.860	0.406	0.077	0.501

^a–b^ Means labeled with no common superscript in the same row differed significantly (*p* < 0.05). ^1^ Data were presented with the means and SEM (n = 12). −LPS, injected with saline; +LPS, injected with LPS; −NG, fed basal diets; +NG, fed basal diets supplemented with 200 mg/kg NG. VH, villus height; CD, crypt depth; VW, villus width; VA, villus area; VH/CD, the VH-to-CD ratio.

**Table 9:** The effect of NG on the relative expression levels of genes related to intestinal barrier function in broilers challenged with LPS ^1^.

Items	−LPS		+LPS		SEM	*p*-Value		
	−NG	+NG	−NG	+NG		LPS	NG	LPS × NG
Duodenum								
*Claudin-1*	1.00	1.15	0.93	1.25	0.060	0.932	0.054	0.481
*Mucin-2*	1.00	0.40	0.82	0.47	0.057	0.509	<0.001	0.150
*ZO-1*	1.00	1.26	0.81	1.08	0.038	0.004	<0.001	0.946
*Occludin*	1.00	1.37	0.80	0.85	0.055	<0.001	0.024	0.084
Jejunum								
*Claudin-1*	1.00	0.97	1.33	1.33	0.061	0.005	0.873	0.905
*Mucin-2*	1.00 ^a^	0.31 ^c^	0.68 ^b^	0.41 ^c^	0.057	0.182	<0.001	0.011
*ZO-1*	1.00	0.95	1.05	1.08	0.036	0.239	0.910	0.593
*Occludin*	1.00	0.95	1.04	0.71	0.055	0.370	0.081	0.202
Ileum								
*Claudin-1*	1.00	0.93	1.14	1.13	0.063	0.200	0.793	0.806
*Mucin-2*	1.00	0.64	0.84	0.67	0.044	0.413	0.002	0.230
*ZO-1*	1.00 ^ab^	0.92 ^b^	0.80 ^b^	1.19 ^a^	0.040	0.636	0.028	0.002
*Occludin*	1.00	1.07	0.96	1.10	0.053	0.932	0.324	0.750

^a–c^ Means labeled with no common superscript in the same row differed significantly (*p* < 0.05). ^1^ Data were presented with the means and SEM (n = 12). −LPS, injected with saline; +LPS, injected with LPS; −NG, fed basal diets; +NG, fed basal diets supplemented with 200 mg/kg NG. *ZO-1*, zonula occludens-1.

**Table 10:** The effect of NG on the relative expression levels of immune response related genes in broiler liver challenged with LPS ^1^.

Items	−LPS		+LPS		SEM	*p*-Value		
	−NG	+NG	−NG	+NG		LPS	NG	LPS × NG
*IL-1β*	1.00	2.53	15.15	15.45	1.299	<0.001	0.558	0.694
*IL-8*	1.00	1.70	18.63	20.06	1.586	<0.001	0.520	0.825
*INOS*	1.00	1.29	13.18	17.55	1.412	<0.001	0.213	0.274
*TNF-α*	1.00	1.18	3.16	3.03	0.183	<0.001	0.907	0.474
*NF-kB*	1.00	1.31	1.20	1.44	0.058	0.131	0.016	0.764
*TLR-4*	1.00 ^b^	1.56 ^a^	0.65 ^c^	0.66 ^c^	0.077	<0.001	0.015	0.018
*IFN-γ*	1.00 ^b^	1.50 ^a^	0.86 ^b^	0.66 ^b^	0.077	<0.001	0.251	0.010

^a–c^ Means labeled with no common superscript in the same row differed significantly (*p* < 0.05). ^1^ Data were presented with the means and SEM (n = 12). −LPS, injected with saline; +LPS, injected with LPS; −NG, fed basal diets; +NG, fed basal diets supplemented with 200 mg/kg NG. *IL-1β*, Interleukin-1β; *IL-8*, Interleukin-8; *iNOS*, Inducible nitric oxide synthase; *TNF-α*, Tumor necrosis factor alpha; *NF-κB*, Nuclear factor kappa-light-chain-enhancer of activated B cells; *TLR4*, Toll-like receptor 4; *IFN-γ*, Interferon gamma.

**Table 11:** The effect of NG on the relative expression levels of immune response related genes in broiler intestine challenged with LPS ^1^.

Items	−LPS		+LPS		SEM	*p*-Value		
	−NG	+NG	−NG	+NG		LPS	NG	LPS × NG
Duodenum								
*IL-1β*	1.00	1.13	2.38	3.29	0.227	<0.001	0.163	0.289
*IL-8*	1.00	0.80	3.44	4.23	0.313	<0.001	0.493	0.259
*INOS*	1.00	1.16	1.15	1.28	0.061	0.281	0.250	0.920
*TNF-α*	1.00	1.18	1.24	1.29	0.049	0.068	0.258	0.491
*NF-kB*	1.00	1.14	1.24	1.12	0.045	0.221	0.931	0.154
*TLR-4*	1.00	1.05	0.59	0.83	0.042	<0.001	0.033	0.159
*IFN-γ*	1.00	0.80	1.95	1.64	0.119	<0.001	0.196	0.781
Jejunum								
*IL-1β*	1.00	1.49	2.96	2.12	0.231	0.004	0.669	0.118
*IL-8*	1.00	0.91	3.76	3.13	0.313	<0.001	0.491	0.606
*INOS*	1.00	0.92	1.66	1.19	0.135	0.088	0.305	0.465
*TNF-α*	1.00 ^b^	1.16 ^ab^	1.41 ^a^	1.07 ^b^	0.051	0.103	0.342	0.011
*NF-kB*	1.00	1.09	1.27	1.10	0.041	0.096	0.623	0.108
*TLR-4*	1.00	0.92	0.85	0.96	0.040	0.521	0.837	0.243
*IFN-γ*	1.00	0.80	2.75	1.77	0.153	<0.001	0.008	0.069
Ileum								
*IL-1β*	1.00	0.84	2.64	2.55	0.200	<0.001	0.694	0.917
*IL-8*	1.00	1.06	1.96	1.97	0.176	0.008	0.925	0.932
*INOS*	1.00	0.84	1.53	1.30	0.133	0.067	0.454	0.890
*TNF-α*	1.00	0.87	1.13	1.25	0.044	0.003	0.971	0.137
*NF-kB*	1.00	1.13	1.26	1.51	0.055	0.002	0.057	0.537
*TLR-4*	1.00	1.06	0.97	1.30	0.057	0.332	0.091	0.233
*IFN-γ*	1.00	1.15	1.93	1.65	0.128	0.004	0.789	0.367

^a–b^ Means labeled with no common superscript in the same row differed significantly (*p* < 0.05). ^1^ Data were presented with the means and SEM (n = 12). −LPS, injected with saline; +LPS, injected with LPS; −NG, fed basal diets; +NG, fed basal diets supplemented with 200 mg/kg NG. *IL-1β*, Interleukin-1β; *IL-8*, Interleukin-8; *iNOS*, Inducible nitric oxide synthase; *TNF-α*, Tumor necrosis factor alpha; *NF-κB*, Nuclear factor kappa-light-chain-enhancer of activated B cells; *TLR4*, Toll-like receptor 4; *IFN-γ*, Interferon gamma.

**Table 12:** The effect of NG on the relative expression levels of liver injury related genes in broiler challenged with LPS ^1^.

Items	−LPS		+LPS		SEM	*p*-Value		
	−NG	+NG	−NG	+NG		LPS	NG	LPS × NG
*MMP-13*	1.00 b	1.40 a	0.60 c	0.34 d	0.088	<0.001	0.591	0.016
*MMP-9*	1.00	1.57	6.47	5.91	0.455	<0.001	0.989	0.276
*XIAP*	1.00	1.82	1.54	2.22	0.113	0.018	<0.001	0.722
*BCL-2*	1.00 b	1.48 a	0.73 c	0.83 bc	0.055	<0.001	<0.001	0.012
*Caspase-3*	1.00	1.24	0.87	1.04	0.057	0.145	0.069	0.759

^a–d^ Means labeled with no common superscript in the same row differed significantly (*p* < 0.05). ^1^ Data were presented with the means and SEM (n = 12). −LPS, injected with saline; +LPS, injected with LPS; −NG, fed basal diets; +NG, fed basal diets supplemented with 200 mg/kg NG. *MMP-13*, Matrix metalloproteinase-13; *MMP-9*, Matrix metalloproteinase-9; *BCL-2*, B-cell lymphoma 2; *XIAP*, X-linked inhibitor of apoptosis protein.

## Data Availability

The original contributions presented in the study are included in the article, further inquiries can be directed to the corresponding author.
